# Sectoral roles in greenhouse gas emissions and policy implications for energy utilization and carbon emissions trading: a case study of Beijing, China

**DOI:** 10.1186/s40064-016-2982-y

**Published:** 2016-08-08

**Authors:** Jianping Ge, Yalin Lei, Qun Xu, Xibo Wang

**Affiliations:** 1 School of Humanities and Economic Management, China University of Geosciences (Beijing), Beijing, 100083 China; 2 Key Laboratory of Carrying Capacity Assessment for Resource and Environment, Ministry of Land and Resources, Beijing, 100083 China; 3 The State Key Laboratory of Management and Control for Complex Systems, Institute of Automation, Chinese Academy of Sciences, Beijing, 100190 China

**Keywords:** Input–output, Sectoral roles, Greenhouse gas emissions, Carbon emissions trading, Energy utilization, Beijing

## Abstract

In this study, a decomposition and emissions matrix is developed to identify the roles (giver or taker) played by the sectors in the greenhouse gas emissions for the economy of Beijing in China. Our results indicate that services were the most important emitter if we consider the total (direct and indirect) emissions. In addition to Construction, Scientific studies and technical services and Finance sectors of services were the largest takers. They have a large role in boosting greenhouse gas emissions throughout the economy of Beijing. As the basis and supporter of production activities, the electricity production and the transportation sectors were the greatest givers. More emphasis should be placed on using clean energy and carbon capture and storage technologies to reduce emissions within these sectors. Based on the roles played by these sectors in greenhouse gas emissions, some policy implications were proposed for energy utilization and carbon emissions trading.

## Background

**Fig. 1:**
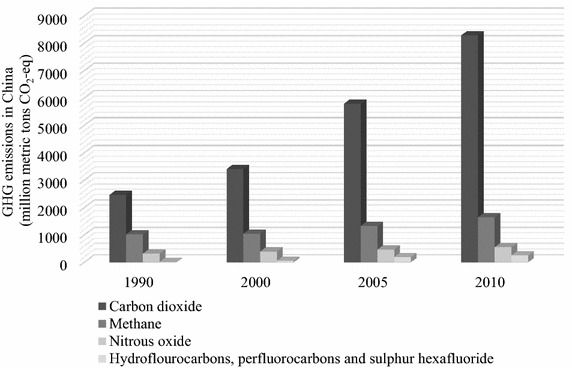
China’s greenhouse gas emissions from 1990 to 2010 (million metric tons of CO_2_-eq)

China has become the second largest economy in the world and is expected to continue to exhibit sustained expansion. However, at the same time, China is the world’s largest emitter of greenhouse gases (GHG) due to domestic coal-dominated energy consumption and the low efficiency of energy use (Leggett 2011). From 1990 to 2010, China’s GHG emissions increased from 3808.3 to 10,728.9 million metric tons of CO_2_ equivalent, as indicated in Fig. 1. Among GHGs, CO_2_, CH_4_ and N_2_O were still the most important pollutions. As is well known, GHG emissions can cause global warming in addition to related natural disasters and losses in public health (Smith et al. 2010; Montzka et al. 2011). However, many people pay more attention to CO_2_, which is only one of the GHG emissions. Non-CO_2_ emissions, such as CH_4_ and N_2_O, also contribute significantly to warming but have not received as much attention (Bousquet et al. 2006; Karakurt et al. 2012).

Because China is the largest CO_2_-emitting country in the world, with a share of 27.50 % in 2012 (OECD 2016), the Chinese government has committed to reducing CO_2_ emissions per unit of gross domestic product (GDP) by 40–50 % of the 2005 level by the year 2020 (Chen and Zhang 2010). Correspondingly, every province (or municipality directly under the central government) faces pressure to reduce local CO_2_ emission per GDP by 10–19.5 % by 2015 according to the “12th Five-Year” GHG emission control program released by the State Council of the People’s Republic of China in 2011. Beijing is the capital of China and one of the world’s largest metropolises with a higher population density, urbanization rate and per capita gross regional product (GRP) compared to the national level, as displayed in Table 1. Economic growth and urbanization increases energy consumption (Shahbaz et al. 2015; Solarin and Lean 2016). In 2012, household energy consumption reached 139.87 million tons of standard coal, accounting for 19.49 % of the total energy consumption of the city of Beijing. Not surprisingly, the remaining energy is consumed by the industrial sectors, of which primary industry accounts for 1.40 %, secondary industry accounts for 33.80 % and tertiary industry is the largest energy consuming sector. Due to the dominance of the energy structure by fossil fuel in Beijing, the high level of energy consumption has resulted in high GHG emissions (Feng et al. 2013; Ge and Lei 2014). According to the “12th Five-Year” GHG emission control program, Beijing’s local CO_2_ emission per GDP is set to decrease by 18 % by 2015.

**Table 1:** General information regarding Beijing and China in 2012. *Source*: Data for Beijing is from BMBS (2013); Data for China is from NBS (2013)

	Population density (person/km^2^)	Per capita GRP/GDP (CNY)	Area (km^2^)	Urbanizaiton level (%)
Beijing	1261.00	87,475.00	16,410.54	86.20
China	141.00	38,459.47	9,596,960.00	52.57

Currently, there is growing concern about climate change and energy-related GHG emissions at various levels in China (Chen and Zhang 2010; Ou et al. 2011; Xi et al. 2011; Liu et al. 2012; Wang et al. 2014; Zhang and Chen 2014; Zhu 2014). Although certain studies have focused on estimating CO_2_ emissions (Guo et al. 2012; Wang et al. 2012; Tian et al. 2013) and GHG emissions in a specific field, such as urban agriculture (Liang et al. 2013) and eco-industrial parks (Liu et al. 2014), there are few systematic studies focusing on the different types of GHG emissions in the city of Beijing. Moreover, because the vast majority of GHG emissions are from production activities, additional efforts are required to understand the different roles of the industrial sectors in different types of GHG emissions, namely, the giver and taker. Based on the different roles played by the sectors in GHG emissions, some recommendations on carbon emissions trading (CET), which is the latest CO_2_ emissions reduction policy, can be proposed for Beijing.

Several studies employ input–output methodology to analyze GHG emission investigations, as input–output analysis can capture both direct and indirect emissions based on the forward and backward linkages between industrial sectors and the effects of final demand changes (Lenzen 1998; Munksgaard and Pedersen 2001; Lenzen et al. 2004; Wiedmann et al. 2007; Wiedmann 2009; Chen and Chen 2011; Ge and Lei 2014). Due to the data accessibility, the majority of studies analyze emissions at the national level (Casler and Rose 1998; Macedo 1998; Machado et al. 2001; Labandeira and Labeaga 2002; Bin and Dowlatabadi 2005; Wihersaari 2005; Butnar and Llop 2007; Mäenpää and Siikavirta 2007; Andrew and Forgie 2008; Alcántara and Padilla 2009; Acquaye and Duffy 2010; Llop and Richard 2013). As the highest GHG emitter, China faces great pressure to reduce emissions while encouraging economic growth. Therefore, a number of studies focusing on the GHG embodiments of China on a national and regional scale have emerged in recent years (Dong et al. 2013; Lindner et al. 2013; Su and Ang 2013; Su et al. 2013; Wang et al. 2013; Zhang 2013; Zhang et al. 2014a, b). However, many authors usually choose CO_2_ as the object of study because of the data accuracy of the emissions data (Wiedmann et al. 2011; Zhang et al. 2014a, b).

This paper attempts to give a full view of the sectoral roles in GHG emissions in the production system, using as its basis the Beijing economy in 2010; it addresses the major GHGs, including CO_2_, CH_4_ and N_2_O, with the most recently released official regional inventory and outlines the corresponding policy implications for CET in Beijing. In this paper, we employ an input–output model to study the generation and transmission of GHG emissions within the production system and to assess the roles played by each sector in which GHG emissions can be found. The rest of the paper is organized as follows. “Methods” section describes the input–output model and its application to the analysis of GHG emissions. “Results” section applies the model to the Beijing economy and identifies the sectors that are givers and the sectors that are takers in the production system. “Discussion” section proposes policy implications for CET in Beijing. Finally, “Conclusions and policy implications” section concludes the study.

## Methods

### Model specification

The input–output model is based on the model developed in Alcántara and Padilla (2009), namely, the input–output subsystem model. We develop certain changes to extend it to the whole production system and use it to analyze the roles played by the sectors in the GHG emissions. The input–output subsystem approach makes it possible to study a limited number of production activities, an individual sector or a group of sectors, which are considered a subsystem that interacts with the rest of the production system (Llop and Richard 2013). Here, we can cite the works of the pioneer and developer of subsystems construction (Sraffa 1960; Harcourt and Massaro 1964; Pasinetti 1973, 1988; Deprez 1990; Scazzieri 1990). Next, the input–output subsystem model is extended to the environmental field (Sánchez-Chóliz and Duarte 2003; Alcántara and Padilla 2009; Butnar and Llop 2011; Llop and Richard 2013; Ge and Lei 2014).

To start the subsystem construction of the model, we assume that the input–output system has N production sectors. These production sectors are divided into the M category and S category, with $1,2,3, \ldots ,{\text{m}}$ sectors belonging to the M subsystem and ${\text{m}} + 1,{\text{m}} + 2, \ldots ,{\text{n}}$ belonging to the ${\text{S}}$ subsystem. Based on the Leontief model, a balance equation can be obtained based on this separation of the accounts as follows:

1 $$\left( {\begin{array}{*{20}c} {A_{MM} } & {A_{MS} } \\ {A_{SM} } & {A_{SS} } \\ \end{array} } \right)\left( {\begin{array}{*{20}c} {x^{M} } \\ {x^{S} } \\ \end{array} } \right) + \left( {\begin{array}{*{20}c} {y^{M} } \\ {y^{S} } \\ \end{array} } \right) = \left( {\begin{array}{*{20}c} {x^{M} } \\ {x^{S} } \\ \end{array} } \right)$$

where matrices A contain technical coefficients of the input–output table, $x^{M}$ and $x^{S}$ denote sectoral production, and $y^{M}$ and $y^{S}$ denote sectoral final demand (including domestic consumption and export). If ${\text{B}} = \left( {I - A} \right)^{ - 1}$, where B represents the Leontief inverse matrices, the expression (1) can be written as:

2 $$\left( {\begin{array}{*{20}c} {A_{MM} } & {A_{MS} } \\ {A_{SM} } & {A_{SS} } \\ \end{array} } \right)\left( {\begin{array}{*{20}c} {B_{MM} } & {B_{MS} } \\ {B_{SM} } & {B_{SS} } \\ \end{array} } \right)\left( {\begin{array}{*{20}c} {y^{M} } \\ {y^{S} } \\ \end{array} } \right) + \left( {\begin{array}{*{20}c} {y^{M} } \\ {y^{S} } \\ \end{array} } \right) = \left( {\begin{array}{*{20}c} {x^{M} } \\ {x^{S} } \\ \end{array} } \right)$$

Solving expression (2), the following Eqs. (3) and (4) can be obtained to reflect the production of the M and S subsystems, respectively.

3 $$A_{MM} B_{MM} y^{M} + A_{MS} B_{SM} y^{M} + A_{MM} B_{MS} y^{S} + A_{MS} B_{SS} y^{S} + y^{M} = x^{M}$$

4 $$A_{SM} B_{MM} y^{M} + A_{SS} B_{SM} y^{M} + A_{SM} B_{MS} y^{S} + A_{SS} B_{SS} y^{S} + y^{S} = x^{S}$$

The matrices A are all decomposed into $A^{D}$ and $A^{0}$, so that ${\text{A}} = A^{D} + A^{0}$. $A^{D}$ are defined as the diagonal matrices for which the main diagonal elements are from the matrices A and the remaining elements are zero, while $A^{0}$ are the matrices for which the main diagonal elements are zero and for which the remaining elements are from matrices A. Let us assume that we are interested in analyzing the S subsystem first. Therefore, we can define $y^{M} = 0$. Then, we can obtain the Eqs. (5) and (6) as follows (Alcántara and Padilla 2009):

5 $$A_{MM}^{D} B_{MS} y^{S} + A_{MM}^{0} B_{MS} y^{S} + A_{MS}^{0} B_{SS} y^{S} + 0 = x_{S}^{M}$$

6 $$A_{SS}^{D} B_{SS} y^{S} + A_{SM}^{0} B_{MS} y^{S} + A_{SS}^{0} B_{SS} y^{S} + y^{S} = x_{S}^{S}$$

where ${\text{x}}_{\text{S}}^{\text{M}}$ indicates the sectors belonging to M subsystem produce commodities or services to meet final demand in the sectors of S subsystem, and ${\text{x}}_{\text{S}}^{\text{S}}$ indicates the production of S needed to cover its own final demand. Equations (5) and (6) involve five components generated by the final demand of S subsystem. The first term ${\text{A}}_{\text{MM}}^{\text{D}} {\text{B}}_{\text{MS}} {\text{y}}^{\text{S}} + {\text{A}}_{\text{MM}}^{0} {\text{B}}_{\text{MS}} {\text{y}}^{\text{S}} + {\text{A}}_{\text{MS}}^{0} {\text{B}}_{\text{SS}} {\text{y}}^{\text{S}}$ or ${\text{x}}_{\text{S}}^{\text{M}}$, which is also shown as Eq. (5), is defined as the spillover component (SOC). The other four components are all included in the left hand side of Eq. (6). The term ${\text{A}}_{\text{SS}}^{\text{D}} {\text{B}}_{\text{SS}} {\text{y}}^{\text{S}}$ denotes the inputs into the S subsectors that are needed to meet their own final demand, namely, the own component (OC). The vector ${\text{y}}^{\text{S}}$ denotes the final demand of S and represents the direct effect of final demand. We call this component the demand volume component (DVC). Additionally, the term ${\text{A}}_{\text{SM}}^{0} {\text{B}}_{\text{MS}} {\text{y}}^{\text{S}}$ indicates inputs produced in S for M to meet the final demand of S. This is the feedback component (FBC). Finally, the term ${\text{A}}_{\text{SS}}^{0} {\text{B}}_{\text{SS}} {\text{y}}^{\text{S}}$ denotes the mutual demand among S branches themselves and is referred to as the intra-sector spillover component (ISC).

For M subsystem, the same assumption and decomposition are employed to obtain five components caused by the final demand of M subsystem.

To obtain sectoral GHG emissions in the above five components, we use matrices $c^{M}$ and $c^{S}$ that contain the emissions coefficients expressed by the emissions per unit of production (tons/10,000 CNY) in the M and S subsystems, respectively. The matrices for the five components have emissions in rows and sectors in columns. Therefore, the emissions associated with the components generated by the final demand of S can be expressed as:

7 $$SOE_{S} = c^{M} \left( {A_{MM}^{D} B_{MS} + A_{MM}^{0} B_{MS} + A_{MS}^{0} B_{SS} } \right)y^{S}$$

8 $$OE_{S} = c^{S} A_{SS}^{D} B_{SS} y^{S}$$

9 $$FBE_{S} = c^{S} A_{SM}^{0} B_{MS} y^{S}$$

10 $$ISE_{S} = c^{S} A_{SS}^{0} B_{SS} y^{S}$$

11 $$DVE_{S} = c^{S} y^{S}$$

Similarly, the emissions associated with the components caused by the final demand of M can be expressed as:

12 $$SOE_{M} = c^{S} \left( {A_{SM}^{0} B_{MM} + A_{SS}^{D} B_{SM} + A_{SS}^{0} B_{SM} } \right)y^{M}$$

13 $$OE_{M} = c^{M} A_{MM}^{D} B_{MM} y^{M}$$

14 $$FBE_{M} = c^{M} A_{MS}^{0} B_{SM} y^{M}$$

15 $$ISE_{M} = c^{M} A_{MM}^{0} B_{MM} y^{M}$$

16 $$DVE_{M} = c^{M} y^{M}$$

Based on the above components for the M and S subsystems, we can draw an emission matrix R between sectors as in Table 2. For any sector belonging to M and S, the matrix includes the emission effect generated by the production of the sector itself and the other sectors in the system. To represent these two emission effects, we decompose matrix R into two matrices, $$R^{D}$$ and $$R^{0}$$, so that $${\text{R}} = R^{D} + R^{0}$$. Here, $$R^{D}$$ is a matrix $$\left( {n \times n} \right)$$ for which the main diagonal contains the elements of R and the other elements are zeros, while $$R^{0}$$$$\left( {n \times n} \right)$$ contains the rest of the elements of R and has zeros in its main diagonal. Therefore, the emission effect generated by the production of a specific sector, namely, sector $${\text{i }}\left( {{\text{i}} \in {\text{n}}} \right)$$, can be expressed by $$R^{D}$$, and the emission effect caused by the other sectors in the system can be expressed by $$R^{0}$$. Moreover, the emission effect caused by the other sectors can be further decomposed into the pull effect and push effect of the specific sector $${\text{i }}\left( {{\text{i}} \in {\text{n}}} \right)$$. The pull effect can be expressed by $$\mathop \sum \nolimits_{j = 1}^{n} R_{ij}^{0}$$, which indicates the GHG emissions generated by the other sectors of the system as a consequence of the final demand of the specific sector $${\text{i }}\left( {{\text{i}} \in {\text{n}}} \right)$$. The push effect can be expressed by $$\sum\nolimits_{i = 1}^{n} {R_{ij}^{0} }$$, which indicates the GHG emissions generated by the specific sector $${\text{i }}\left( {{\text{i}} \in {\text{n}}} \right)$$ due to the final demand of the other sectors of the system. A specific sector is defined as the giver when the pull effect is larger than the push effect or as the taker if the push effect is more significant compared with the pull effect in the production system.

**Table 2:** Emission matrix R based on emission components

	M	S
M	OE_m_ + FBE_m_ + ISE_m_	SOE_s_
S	SOE_m_	OE_s_ + FBE_s_ + ISE_s_

### Data

The input–output data for Beijing used in this study are from the input–output extension table of Beijing for 2010 (BMBS 2012). The level of disaggregation in the input–output extension table is 40 sectors. Detailed sectoral information is displayed in Appendix Table 14. However, the industry classification is somewhat different between China and abroad. Because Construction (sector 26) is a quite important sector in this study and the China’s definition of construction work is inconsistent with ISIC Rev.4 (International Standard Industrial Classification of All Economic Activities, Rev.4) and NAICS 2007 (North American Industry Classification System, 2007), the definition of Construction (sector 26) needs to be explained. Construction (sector 26) in China indicates the construction of buildings or engineering projects (e.g., highways) and includes new work, additions, and alterations. However, it excludes maintenance and repairs, which is different with ISIC Rev.4 and NAICS 2007.

The input–output extension table of Beijing for 2010 provided two kinds of trading information between Beijing local and China domestic market as well as between Beijing local and foreign market. Based on the above information, the sectoral GHG emissions from local, domestic and foreign markets can be estimated, which can be used to divide the responsibilities for the GHG emissions between different markets. However, focusing on the sectoral role in the GHG emissions with implications to Beijing’s CET, we adopted a single-region assumption in our analysis as other studies (Chen and Chen 2010; Zhou et al. 2010; Butnar and Llop 2011; Guo et al. 2012). This assumption indicates that for both domestic imports and foreign imports, imported commodities have the same emission intensities as local ones (Guo et al. 2012). Though the energy-economic transactions between Beijing and other regions would affect the responsibilities for the GHG emissions, the CET only acts on the sectors in Beijing and has not extended to the national level. Therefore, a single-region assumption is suitable to the current GHG emissions accounting and CET for Beijing. Our analysis has already obtained the sectoral roles in the GHG emissions that can provide some policy implications on the Beijing’s CET.

We focus on the three groups of greenhouse gases that are CO_2_, N_2_O and CH_4_. GHG emissions per unit of output are estimated based on sectoral energy consumption and a carbon emission factor (determined by fuel type) and is given by the following expression:

17 $$C_{n}^{m} = \frac{{\sum E_{i} f_{i}^{m} }}{{X_{n} }}$$

where $C_{n}^{m}$ is the mth GHG emissions per unit of output in the nth sector of the production system, $E_{i}$ is energy consumption of the ith fuel type in the nth sector, $f_{i}^{m}$ is the mth GHG emission factor of the ith fuel type, and $X_{n}$ is the output of the nth sector.

The GHG emission factor for the eight detailed types of energy fuel $f_{i}^{m}$ can be calculated based on the 2006 IPCC Guidelines for National Greenhouse Gas Inventories, displayed in Table 3. The data on energy consumption are from the Beijing Statistical Yearbook 2011 (BMBS 2011). Based on the availability of data from the above two sources, the GHG emissions per unit output in the sectors of the production system can be obtained, as displayed in Table 4.

**Table 3:** GHG emissions factor in China

	Unit	CO_2_ emissions (kg)	N_2_O emissions (kg)	CH_4_ emissions (kg)
Coal	kg	2.05	3.14E−05	2.09E−05
Coke	kg	3.05	4.27E−05	2.85E−05
Gasoline	kg	3.02	2.59E−05	1.29E−04
Kerosene	kg	3.10	2.59E−05	1.29E−04
Diesel oil	kg	3.16	2.56E−05	1.28E−04
Fuel oil	kg	3.24	2.51E−05	1.26E−04
Liquefied petroleum gases	kg	3.17	5.02E−06	5.02E−05
Natural gas	m^3^	2.19	3.90E−06	3.90E−05

**Table 4:** Sectoral GHG emissions per unit of output for the year 2010 (tons/10,000 CNY)

Sector code	CO_2_ emission	N_2_O emission	CH_4_ emission
1	0.394	5.33E−06	7.12E−06
2	0.005	6.85E−08	7.41E−08
3	0.330	2.49E−06	1.24E−05
4	3.981	5.77E−05	4.05E−05
5	2.149	2.79E−05	3.62E−05
6	0.206	2.82E−06	2.65E−06
7	0.235	3.38E−06	3.05E−06
8	0.174	2.50E−06	2.36E−06
9	0.081	1.01E−06	1.60E−06
10	0.171	2.09E−06	2.67E−06
11	0.381	2.37E−06	1.21E−05
12	0.226	3.17E−06	2.99E−06
13	1.089	1.52E−05	1.50E−05
14	0.075	4.36E−07	1.26E−06
15	0.081	8.50E−07	1.56E−06
16	0.055	6.73E−07	9.63E−07
17	0.042	4.15E−07	7.14E−07
18	0.029	3.48E−07	4.96E−07
19	0.004	2.23E−08	9.93E−08
20	0.015	1.32E−07	4.31E−07
21	0.072	9.41E−07	1.04E−06
22	0.316	4.58E−06	4.38E−06
23	1.457	1.87E−05	1.72E−05
24	0.109	2.31E−07	2.02E−06
25	0.086	4.79E−07	1.80E−06
26	0.064	5.82E−07	2.19E−06
27	0.729	1.75E−05	4.55E−05
28	0.006	4.11E−07	1.24E−06
29	0.032	2.26E−06	6.89E−06
30	0.190	1.95E−06	4.17E−05
31	0.004	2.99E−07	9.28E−07
32	0.204	2.85E−06	6.14E−06
33	0.039	1.22E−06	3.27E−06
34	0.020	8.63E−07	2.61E−06
35	0.130	6.30E−06	1.52E−05
36	0.206	4.09E−06	1.00E−05
37	0.181	2.95E−06	8.63E−06
38	0.061	9.37E−07	2.75E−06
39	0.038	1.14E−06	3.78E−06
40	0.058	2.83E−06	8.56E−06

As indicated in Table 4, the sectors had different levels of GHG emissions per unit of output, in which the CO_2_ emissions per unit of output were in the range of 0.004 tons and 3.981 tons, the N_2_O emissions per unit of output were between 2.23E−08 tons and 5.77E−05 tons, and the CH_4_ emissions per unit of output ranged from 7.41E−08 tons to 4.55E−05 tons. The CO_2_ and N_2_O emissions had a similar distribution of sectoral emissions. Among the sectors, the top three sectors with the highest levels of CO_2_ and N_2_O emissions per unit of output were Mining and processing of metal ores (sector 4), Mining and processing of nonmetal ores and other ores (sector 5) and Production and distribution of electricity and heat (sector 23). Compared with CO_2_ and N_2_O emissions, Transportation, storage, posts and telecommunications (sector 27), Hotel and restaurants (sector 30) and Mining and processing of metal ores (sector 4) were the largest CH_4_ emitters in terms of per unit of output. It can be observed that manufacturing had a higher level of CO_2_ and N_2_O emissions, while services played an important role in CH_4_ emissions in terms of per unit of sectoral production. This may be because services, such as Transportation, storage, posts and telecommunications, consumed much more gasoline, kerosene and diesel oil compared to manufacturing, and these types of energy fuels had high CH_4_ emission factors. Although the GHG emissions intensity per unit of output have been obtained, it is necessary to determine the sectors that were givers and the sectors that were takers in the production system to establish the emission reduction path and policy implications.

## Results

This section reports three types of information: first, the sectoral direct emissions for Beijing in 2010; second, total (direct and indirect) emissions generated by the final demand of sectors; third, the decomposition of the total emissions generated by the final demand of sectors into the pull effect and the push effect in the production system in addition to the sectoral role in the emissions including the givers and the takers.

### Direct emissions

Table 5 displays the sectoral direct emissions of CO_2_, N_2_O and CH_4_ based on the energy consumption in the process of commodity production or the providing of services. To compare different GHG emissions in the same dimension and obtain the total direct GHG emission, the emissions in CO_2_-equivalent units expressed by the global warming potential (GWP) value from the IPCC Fifth Assessment Report (AR5) (IPCC 2013) are adopted, as listed in Appendix Table 15.

**Table 5:** Sectoral direct emissions (thousand tons CO_2_-eq) for Beijing in 2010

Sectoral code	CO_2_ emission	N_2_O emission	CH_4_ emission
1	1291.61	4.64	0.65
2	28.42	0.11	0.01
3	577.29	1.15	0.61
4	12979.62	49.89	3.70
5	99.16	0.34	0.05
6	1507.84	5.48	0.54
7	190.69	0.73	0.07
8	249.57	0.95	0.09
9	78.35	0.26	0.04
10	425.86	1.38	0.19
11	2423.69	4.00	2.15
12	2609.82	9.71	0.97
13	4418.46	16.31	1.70
14	284.00	0.44	0.13
15	233.86	0.65	0.13
16	613.08	1.97	0.30
17	947.20	2.49	0.45
18	211.27	0.67	0.10
19	100.18	0.13	0.06
20	36.65	0.08	0.03
21	87.24	0.30	0.04
22	39.76	0.15	0.02
23	31525.92	107.31	10.45
24	171.15	0.10	0.09
25	42.53	0.06	0.02
26	2035.43	4.89	1.94
27	19309.07	122.99	33.77
28	176.74	3.26	1.04
29	1190.64	22.37	7.21
30	1998.68	5.45	12.31
31	127.84	2.63	0.86
32	3689.24	13.65	3.11
33	1506.90	12.55	3.57
34	793.94	9.09	2.90
35	300.91	3.87	0.99
36	465.30	2.45	0.63
37	1635.54	7.04	2.18
38	494.34	2.02	0.63
39	288.12	2.27	0.80
40	795.66	10.32	3.29
Total	95981.58	434.18	97.82

Among these three kinds of GHG emissions, CO_2_ emissions were the largest component of the total GHG emissions that lead to climate warming, followed by the N_2_O and CH_4_ emissions. Specifically, CO_2_ emissions, N_2_O emissions and CH_4_ emissions accounted for 99.45, 0.45 and 0.10 %, respectively.

In terms of sectoral emissions, Production and distribution of electricity and heat (sector 23) was the largest emitter of CO_2_, followed by Transportation, storage, posts and telecommunications (sector 27) and Mining and processing of metal ores (sector 4). These three activities accounted for 66.49 % of the total CO_2_ emissions. This result is consistent with expectations and may be related to the following three reasons: First, our electricity generation is still heavily dependent on coal; second, transportation still mainly uses fossil fuels and has not promoted ethanol fuel or other clean energy on a large-scale; third, energy efficiency in the transportation and electricity sectors is low (Zhang et al. 2011; Liu et al. 2011).

Unlike in the case of CO_2_ emissions, Transportation, storage, posts and telecommunications (sector 27) was the largest emitter of N_2_O and CH_4_. This may be because one of the main N_2_O and CH_4_ emission sources is fuel combustion in transportation in China (Zhang et al. 2014a, b). The emissions of N_2_O and CH_4_ from Transportation, storage, posts and telecommunications (sector 27) in 2010 were approximately 28.33 and 34.52 % of the total value of N_2_O and CH_4_ emissions, respectively. This result supports the control policy issued by the Beijing Municipal Government on the growth of the ownership of cars powered by fossil fuels and the promotion policy on electric vehicles (EV) and buses.

Because direct emissions are concentrated in economic sectors that deliver intermediate goods and services to other sectors, the policy makers should take into account emission trade in intermediates and not pay attention solely to sectoral direct emissions (Llop and Richard 2013). Therefore, the GHG emissions generated by the final demand of sectors need to be decomposed to grasp the channels of the sectoral GHG emissions and to understand the role of emissions.

### Total emissions generated by the final demand of sectors

**Fig. 2:**
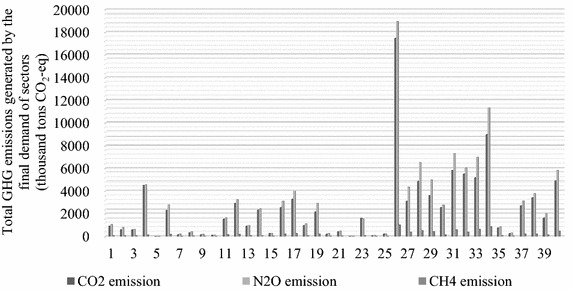
GHG emissions generated by the final demand of sectors (thousand tons CO_2_-eq) for Beijing in 2010

Figure 2 displays the GHG emissions generated by the final demand of sectors in 2010. Specifically, for all three types of GHG emissions, CO_2_, N_2_O and CH_4_, Construction (sector 26) was the largest emitter. In general, the service sectors generated more GHG emissions than the manufacturing sectors (excluding Construction) and the agricultural sector. Though the service sectors were not large direct emitters of GHGs, they played an enormous role in pulling the manufacturing and the agricultural production (Alcántara and Padilla 2009; Llop and Richard 2013; Ge and Lei 2014).

The total CO_2_ emissions were 99,895.20 thousand tons, of which 17.47 % were caused by Construction, 8.99 % were generated by Scientific studies and technical services (sector 34), and 5.84 % were caused by Finance (sector 31). In addition, the emissions generated by the other thirteen sectors (sectors 4, 12, 16, 17, 27, 28, 29, 30, 32, 33, 37, 38, and 40) are above the average, most of which belong to services. Services (sectors 27–40) explain 53.36 % of the total CO_2_ emissions caused by the final demand of all sectors. It is somewhat unexpected and also reflected in the N_2_O and CH_4_ emissions that Mining and processing of nonmetal ores and other ores (sector 5) exhibited the smallest contribution to climate change. This may be because Mining and processing of nonmetal ores and other ores (sector 5) was not closely linked with the other sectors and sustained a small impact from the final demand of the other sectors.

Similar to CO_2_ emissions, as Table 5 indicates, Construction (sector 26) was the largest emitter of N_2_O emissions, with approximately 16.03 % (18,941.03 thousand tons CO_2_-eq) of the total N_2_O emissions in 2010. The emissions caused by the other fourteen sectors (sectors 4, 12, 16, 17, 27, 28, 29, 31, 32, 33, 34, 37, 38, and 40) are all above the average, ten of which belong to services.

For CH_4_ emissions, Construction (sector 26) was still the largest emitter, as is the case for CO_2_ and N_2_O emissions, followed by Scientific studies and technical services (sector 34) and Tenancy and commercial services (sector 33). Jointly, these three activities account for approximately one-third of total CH_4_ emissions. The emissions generated by the other eleven sectors (sectors 16, 17, 19, 27, 28, 29, 31, 32, 37, 38, and 40) are all above the average, eight of which belong to services, accounting for 57.95 % of the total CH_4_ emissions.

### Pull effect, push effect and the role in emissions of the sectors

**Fig. 3:**
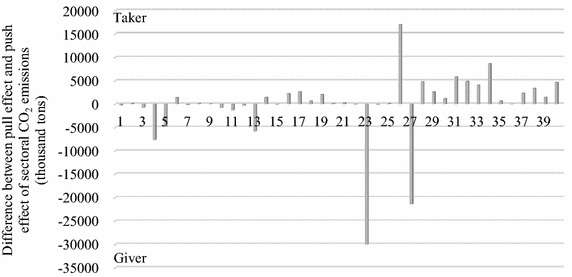
Pull and push effect (thousand tons) and the sectoral role in CO_2_ emissions

**Fig. 4:**
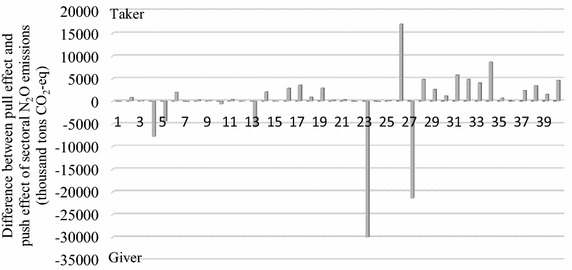
Pull and push effect (thousand tons CO_2_-eq) and the sectoral role in N_2_O emissions

**Fig. 5:**
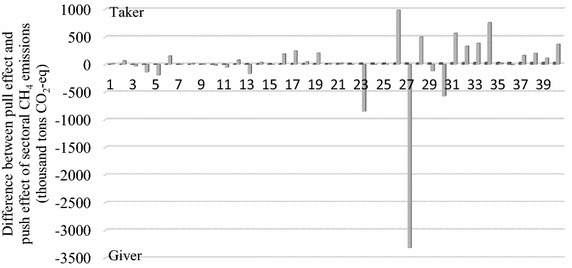
Pull and push effect (thousand tons CO_2_-eq) and the sectoral role in CH_4_ emissions

Figures 3, 4 and 5 indicate the pull effect and push effect of sectors and the sectoral role in CO_2_, N_2_O and CH_4_ emissions. The ‘Difference’ in the above figures is obtained by the value of the pull effect minus the value of the push effect, by which we defined the role of each sector in the GHG emissions. A sector defined by the ‘Giver’ role indicates the total emissions generated by the other sectors of the Beijing economy to meet its own final demand, namely, the pull effect, and is less than the emissions caused by the ‘Giver’ sector itself to meet the final demand of the other sectors, namely, the push effect. On the contrary, a sector defined by the ‘Taker’ role means its pull effect is larger than its push effect.

### CO_2_ emissions

Figure 3 indicates the pull effect and push effect of sectors and the sectoral role in CO_2_ emissions.

Not surprisingly, Construction (sector 26) was the largest taker for CO_2_ emissions. The pull effect of Construction for the CO_2_ emissions of the whole Beijing economy was 17,400.37 thousand tons, while the push effect was 375.42 thousand tons, which is only 2.16 % of the pull effect. As Table 6 indicates, Manufacture of non-metallic mineral products (sector 13) suffered the largest pull effect from Construction and emitted 4221.35 thousand tons of CO_2_ to meet the final demand of Construction. Large pull effects were also given to Mining and processing of nonmetal ores and other ores (sector 5), Production and distribution of electricity and heat (sector 23), Mining and processing of metal ores (sector 4) and Transportation, storage, posts and telecommunications (sector 27). The total CO_2_ emissions of the above five sectors was 15,411.87 thousand tons, accounting for 88.57 % of the total pull effect of Construction. The majority of pull effects were applied to these five sectors because Construction requires metals, minerals (e.g., cement and concrete), openings, plastics, timber, fuel (e.g., mains gas and grid electricity), and transport to produce a building or a house, which causes the CO_2_ emissions of the corresponding sector (Monahan and Powell 2011). Furthermore, though the sectors Manufacture of non-metallic mineral products (sector 13) and Mining and processing of nonmetal ores and other ores (sector 5) emitted CO_2_ to meet the final demand of the other sectors, more than half of the CO_2_ emissions are caused by the Construction demand, accounting for 64.30 and 63.84 % of their total CO_2_ emissions. In terms of Mining and processing of metal ores (sector 4) and Transportation, storage, posts and telecommunications (sector 27), as large emitters of CO_2_ emissions, the emissions of these two sectors generated by the final demand for Construction accounted for 30.17 and 10.88 % of their own total emissions, respectively. Thus, the pull effect of Construction was an important factor contributing to the classification of the sectors Mining and processing of metal ores (sector 4) and Transportation, storage, posts and telecommunications (sector 27) as ‘Giver’.

**Table 6:** Pull effect of the largest three takers in CO_2_ emissions

Sector defined as ‘Taker’	Top five receiver of pull effect	Sector code	Pull effect on the receivers (thousand tons)	Proportion in pull effect	Proportion in the receiver’s emission (%)
Construction	Manufacture of non-metallic mineral products	13	4221.35	24.26	64.30
	Mining and processing of nonmetal ores and other ores	5	3096.57	17.80	63.84
	Production and distribution of electricity and heat	23	2863.14	16.45	9.37
	Mining and processing of metal ores	4	2776.42	15.96	30.17
	Transportation, storage, posts and telecommunications	27	2454.39	14.11	10.88
Scientific studies and technical services	Transportation, storage, posts and telecommunications	27	2821.57	31.43	12.51
	Production and distribution of electricity and heat	23	2632.46	29.33	8.61
	Mining and processing of metal ores	4	693.54	7.73	7.54
	Manufacture of non-metallic mineral products	13	500.92	5.58	7.63
	Chemical industry	12	334.56	3.73	11.44
Finance	Production and distribution of electricity and heat	23	2245.54	38.49	7.35
	Transportation, storage, posts and telecommunications	27	1883.95	32.30	8.35
	Processing of petroleum, cokiing, processing of nuclear fuel	11	209.97	3.60	8.05
	Real estate trade	32	195.07	3.34	37.20
	Tenancy and commercial services	33	177.81	3.05	18.13

In addition, there are twenty-four other sectors defined as ‘Taker’. The second and third largest takers were the sectors Scientific studies and technical services (sector 34) and Finance (sector 31), for which the differences between the pull effect and the push effect were 8661.27 thousand tons and 5793.40 thousand tons, respectively. The majority of the pull effects of these twenty-four takers were imposed on the sectors Production and distribution of electricity and heat (sector 23) and Transportation, storage, posts and telecommunications (sector 27). Though services (except sector 27) have small direct CO_2_ emissions, their demand for electricity indirectly stimulates CO_2_ emissions. Specifically, the final demand of the service sectors 28, 30–32, 35 and 37–40 led to 48.19 % of the total CO_2_ emitted by Production and distribution of electricity and heat (sector 23). Because the development of services relies on electricity, to reduce CO2 emissions, the government should focus on improving power conversion efficiency and on the adoption of clean resources for generating electricity such as solar power and wind power. Commercial development and the rise of technical services drive the development of transportation and thus increase the emission of CO_2_: Sectors 29, 33 and 34 caused 29.04 % of the total CO_2_ emissions of sector 27. The Beijing government is promoting services as an important part of future economic development, which is inseparable from the development of transportation. To control the CO2 caused by the final demand of services, the use of clean energy (e.g., bio-ethanol) should be widely applied in transportation (Ge et al. 2014). In addition to services, although the manufacturing sectors were also dependent on electricity and transportation, their pull effects were not large, only accounting for 5.16 and 8.42 % of the total emission generated by sector 23 and sector 27, respectively. With raw materials input, the activities related to the manufacture of metal products, machinery and equipment led to an increase of 26.88 % in CO_2_ emissions generated by Mining and processing of metal ores (sector 4). Similarly, the activities related to clothing production increased the emissions of Manufacture of textiles (sector 7) by 42.35 %.

In terms of the ‘Giver’ side, as Fig. 3 indicates, the largest giver was Production and distribution of electricity and heat (sector 23), followed by the sectors Transportation, storage, posts and telecommunications (sector 27) and the Mining and processing of metal ores (sector 4). This result is in accordance with the above analysis on the ‘Taker’ side. Table 8 indicates that the greatest push effect of CO_2_ emissions generated by Production and distribution of electricity and heat (sector 23) was given to Real estate trade (sector 32) and Construction sector (sector 26), accounting for 10.88 and 8.94 % of the total emission of sector 23, respectively. This may be because rapid development of real estate and construction in Beijing consumed a large quantity of electricity through the equipment for construction and property services. The above reason can be deduced and verified by the following fact that the housing construction area in 2010 increased by 90.95 % compared to that in 2005, and correspondingly, the electricity consumed by construction works increased by 3.90 % in the same period (BMBS 2011). Additionally, Production and distribution of electricity and heat (sector 23) also created the CO_2_ emissions generated to meet the final demand of the service sectors 31, 34 and 40. However, the push effects for sector 23 on the manufacturing sectors were not large, accounting for 0.1 to 5 % of the total. The push effects of Transportation, storage, posts and telecommunications (sector 27) and Mining and processing of metal ores (sector 4) were both to a large extent attributed to Construction (sector 26) and Scientific studies and technical services (sector 34), as indicated by Table 7. The difference between these two large givers is that the push effect of Transportation, storage, posts and telecommunications was mainly explained by the services sectors, including sectors 33, 31 and 29, while the push effect of Mining and processing of metal ores was primarily explained by the manufacturing sectors, including sectors 14, 16 and 17.

**Table 7:** Push effect of the largest three ‘Givers’ in CO_2_ emissions

Sector defined as ‘Giver’	Top five receiver of push effect	Sector code	Push effect on the receivers (thousand tons)	Proportion in push effect (%)
Production and distribution of electricity and heat	Real estate trade	32	3483.99	10.88
	Construction	26	2863.14	8.94
	Scientific studies and technical services	34	2632.46	8.22
	Public manage and social organization	40	2261.98	7.07
	Finance	31	2245.54	7.01
Transportation, storage, posts and telecommunications	Scientific studies and technical services	34	2821.57	11.52
	Construction	26	2454.39	10.02
	Tenancy and commercial services	33	2052.99	8.38
	Finance	31	1883.95	7.69
	Wholesale trade and retail trade	29	1675.63	6.84
Mining and processing of metal ores	Construction	26	2776.42	23.09
	Smelting and pressing of metals	14	1939.92	16.14
	Scientific studies and technical services	34	693.54	5.77
	Manufacture of general and special purpose machinery	16	565.97	4.71
	Manufacture of trans port equipment	17	412.04	3.43

### N_2_O emissions

Figure 4 indicates the pull effect and push effect of sectors and the sectoral role in N_2_O emissions.

Similar to the role played by the sectors in CO_2_ emissions, the largest taker was Construction (sector 26), followed by Scientific studies and technical services (sector 34) and Finance (sector 31). The leading giver was Production and distribution of electricity and heat (sector 23), followed by the sectors Transportation, storage, posts and telecommunications (sector 27) and Mining and processing of metal ores (sector 4).

For a closer analysis on the ‘Taker’, Table 8 has been established to illustrate the pull effect of the largest three takers. It can be seen that these three takers exerted a great pull effect on the sectors Transportation, storage, posts and telecommunications (sector 27) and Production and distribution of electricity and heat (sector 23), which were major fossil energy consumers and the main sources of N_2_O emissions (Zhang et al. 2014a, b). In addition, the proportion of the pull effect on sectors 27 and 23 together with the total pull effect of the three takers were 35.91, 63.62 and 72.01 % for the sectors Construction, Scientific studies and technical services and Finance, respectively. This may explain why these three takers were the largest emitters of N_2_O caused by their final demand. The mining-related sectors such as Mining and processing of metal ores (sector 4) were also N_2_O intensive because they consumed a great deal of electricity and diesel in the stages of mining and mineral processing (Norgate and Haque 2010), which may partly contribute to Construction (sector 26) and Scientific studies and technical services (sector 34) being the largest takers through the pull effect on the mining-related sectors. Though these three sectors were not the largest direct emitters of N_2_O, they played an important role in boosting energy-related sector emissions, the pull effect of which accounted for 4.84–64.30 % of the emissions generated by the effect receivers in the production system, as Table 8 indicates.

**Table 8:** Pull effect of the sectors defined by ‘Taker’ in N_2_O emissions

Sector defined as ‘Taker’	Top five receiver of pull effect	Sector code	Pull effect on the receivers (thousand tons CO2-eq)	Proportion in pull effect (%)	Proportion in the receiver’s emission (%)
Construction	Transportation, storage, posts and telecommunications	27	4189.64	22.12	10.88
	Manufacture of non-metallic mineral products	13	4176.98	22.05	64.30
	Mining and processing of metal ores	4	2860.16	15.10	30.17
	Mining and processing of nonmetal ores and other ores	5	2853.28	15.06	63.84
	Production and distribution of electricity and heat	23	2611.78	13.79	9.37
Scientific studies and technical services	Transportation, storage, posts and telecommunications	27	4816.41	42.45	12.51
	Production and distribution of electricity and heat	23	2401.35	21.17	8.61
	Mining and processing of metal ores	4	714.46	6.30	7.54
	Wholesale trade and retail trade	29	671.16	5.92	13.01
	Manufacture of non-metallic mineral products	13	495.65	4.37	7.63
Finance	Transportation, storage, posts and telecommunications	27	3215.89	43.99	8.35
	Production and distribution of electricity and heat	23	2048.40	28.02	7.35
	Tenancy and commercial services	33	396.76	5.43	18.13
	Wholesale trade and retail trade	29	249.50	3.41	4.84
	Real estate trade	32	193.49	2.65	37.20

In terms of the ‘Giver’, the largest three givers, which were sectors 23, 27 and 4, emitted 5013.13 thousand tons CO_2_-eq, 9006.05 thousand tons CO_2_-eq and 3574.62 thousand tons CO_2_-eq, respectively, to meet the final demand of Construction (sector 26) and Scientific studies and technical services (sector 34), as Table 9 indicates. Specifically, Production and distribution of electricity and heat (sector 23) emitted 3178.12 thousand tons CO_2_-eq N_2_O as a consequence of the Real estate trade (sector 32). This may be related to rapid urbanization, population concentration and the investment expansion of the real estate trade in Beijing after 2000 that led to an increase in electricity consumption by urban residents. In addition, sector 23 also undertook emission reduction pressure caused by the final demand growth of the sectors Public manage and social organization (sector 40) and Finance (sector 31). In addition to sectors 26 and 34, to support the development of the sectors Tenancy and commercial services (sector 33), Finance (sector 31) and Wholesale trade and retail trade (sector 29), Transportation, storage, posts and telecommunications (sector 27) contributed 9580.63 thousand tons CO_2_-eq N_2_O, totaling a third of the sectoral push effect on climate warming. In terms of Mining and processing of metal ores (sector 4), sectors 14, 16 and 17 were major receivers of the push effect because they were the important downstream industry of sector 4 and used metal ores as the intermediate input directly in the production process.

**Table 9:** Push effect of the largest three ‘Givers’ in N_2_O emissions

Sector defined as ‘Giver’	Top five receiver of push effect	Sector code	Push effect on the receivers (thousand tons CO2-eq)	Proportion in push effect (%)
Production and distribution of electricity and heat	Real estate trade	32	3178.12	10.88
	Construction	26	2611.78	8.94
	Scientific studies and technical services	34	2401.35	8.22
	Public manage and social organization	40	2063.39	7.07
	Finance	31	2048.40	7.01
Transportation, storage, posts and telecommunications	Scientific studies and technical services	34	4816.41	16.49
	Construction	26	4189.64	14.35
	Tenancy and commercial services	33	3504.45	12.00
	Finance	31	3215.89	11.01
	Wholesale trade and retail trade	29	2860.29	9.79
Mining and processing of metal ores	Construction	26	2860.16	9.79
	Smelting and pressing of metals	14	1998.42	6.84
	Scientific studies and technical services	34	714.46	2.45
	Manufacture of general and special purpose machinery	16	583.04	2.00
	Manufacture of transport equipment	17	424.47	1.45

### CH_4_ emissions

Figure 5 demonstrates the sectoral role played in CH_4_ emissions in the production system. Construction (sector 26) was the largest taker in CH_4_ emissions, followed by the sectors Scientific studies and technical services (sector 34) and Finance (sector 31). In terms of ‘Giver’, the largest contributors were the sectors Transportation, storage, posts and telecommunications (sector 27), Production and distribution of electricity and heat (sector 23) and Hotel and restaurants (sector 30).

With respect to the ‘Taker’, Construction (sector 26), Scientific studies and technical services (sector 34) and Finance (sector 31) were the largest takers. In Beijing, the notable sources for direct CH_4_ emissions include transportation (34.52 %), catering (12.58 %) and electricity generation (10.68 %), as Table 5 indicates. Because of the strong sector linkages to transportation, the production activities associated with the sectors Construction (sector 26), Scientific studies and technical services (sector 34) and Finance (sector 31) caused a great deal of CH_4_ emissions, of which the pull effects on Transportation, storage, posts and telecommunications (sector 27) accounted for 10.88, 12.51 and 8.35 % of the total emissions generated by sector 27, respectively. A similar mechanism holds for the largest three takers, sectors 26, 34 and 31, and the sectors Production and distribution of electricity and heat (sector 23) and Hotel and restaurants (sector 30), as Table 10 indicates.

**Table 10:** Pull effect of the sectors defined by ‘Taker’ in CH_4_ emissions

Sector defined as ‘Taker’	Top five receiver of pull effect	Sector code	Pull effect on the receivers (thousand tons CO_2_-eq)	Proportion in pull effect (%)	Proportion in the receiver’s emission (%)
Construction	Transportation, storage, posts and telecommunications	27	369.17	36.21	10.88
	Manufacture of non-metallic mineral products	13	139.70	13.70	64.30
	Mining and processing of nonmetal ores and other ores	5	125.47	12.31	63.84
	Production and distribution of electricity and heat	23	81.59	8.00	9.37
	Wholesale trade and retail trade	29	70.37	6.90	13.19
Scientific studies and technical services	Transportation, storage, posts and telecommunications	27	424.40	49.44	12.51
	Hotel and restaurants	30	97.52	11.36	13.56
	Production and distribution of electricity and heat	23	75.02	8.74	8.61
	Wholesale trade and retail trade	29	69.44	8.09	13.01
	Processing of petroleum, cokiing, processing of nuclear fuel	11	17.50	2.04	8.78
Finance	Transportation, storage, posts and telecommunications	27	283.37	48.21	8.35
	Hotel and restaurants	30	89.77	15.27	12.48
	Production and distribution of electricity and heat	23	63.99	10.89	7.35
	Tenancy and commercial services	33	36.19	6.16	18.13
	Wholesale trade and retail trade	29	25.81	4.39	4.84

In terms of the ‘Giver’, Transportation, storage, posts and telecommunications (sector 27) emitted the greatest amount of CH_4_ pollutants because transportation was fueled by petroleum products (e.g., gasoline, diesel oil and fuel oil) and played an important role in the rapidly growing economy (Button and Taylor 2000). To meet the final demand of sectors 34, 26, 33, 31 and 29, as indicated by Table 11, Transportation, storage, posts and telecommunications (sector 27) emitted 1637.77 thousand tons CO_2_-eq of CH_4_, accounting for 44.44 % of the push effects of sector 27 on CH_4_ emissions. As the second largest giver, Production and distribution of electricity and heat (sector 23) emitted 384.35 thousand tons CO_2_-eq of CH_4_ to satisfy the final demand of sectors 32, 26, 34, 40 and 31. Though the CH_4_ emission intensity of sector 23 was not large, electricity generation played a very important role in the production of goods and services, the basis of nearly the entire economy (Apergis and Payne 2011). Therefore, the total push effect of sector 23 on CH_4_ emissions was great. The other important giver was Hotel and restaurants (sector 30). The rapid development of services (e.g., tourism) and the change in the residential lifestyle caused by urbanization in Beijing in recent years has promoted the development of hotels and restaurants. As a CH_4_ emissions-intensive sector, Hotel and restaurants (sector 30) emitted 436.99 thousand tons CO_2_-eq of CH_4_ to meet the final demand of sectors 33, 34, 40, 31 and 28, as indicated by Table 11.

**Table 11:** Push effect of the largest three ‘Givers’ in CH_4_ emissions

Sector defined as ‘Giver’	Top five receiver of push effect	Sector code	Push effect on the receivers (thousand tons CO_2_-eq)	Proportion in push effect (%)
Transportation, storage, posts and telecommunications	Scientific studies and technical services	34	424.40	11.52
	Construction	26	369.17	10.02
	Tenancy and commercial services	33	308.79	8.38
	Finance	31	283.37	7.69
	Wholesale trade and retail trade	29	252.04	6.84
Production and distribution of electricity and heat	Real estate trade	32	99.28	10.88
	Construction	26	81.59	8.94
	Scientific studies and technical services	34	75.02	8.22
	Public manage and social organization	40	64.46	7.07
	Finance	31	63.99	7.01
Hotel and restaurants	Tenancy and commercial services	33	103.37	14.18
	Scientific studies and technical services	34	97.52	13.38
	Public manage and social organization	40	91.09	12.49
	Finance	31	89.77	12.31
	Information transmission, computer services and software	28	55.24	7.58

## Discussion

### Current CET in Beijing

Currently, the Beijing municipal government has formulated a series of policies on energy saving and emissions reduction, such as ‘Beijing’s 12th five-year national action plan on energy conservation and emission reduction’ (2011). Especially for carbon reduction targets, the government started CET in the Beijing area in November 2013 and implemented certain trial policies, e.g., ‘Beijing’s carbon emissions trading management approach (trial)’ (2014). The roles of the sectors in GHG emissions may be helpful in improving the fairness and enforceability of the policies for CET.

Beijing was identified as one of the first group of CET pilot cities by the National Development and Reform Commission of China in 2011. The official opening of CET began in the Beijing Environment Exchange on 28th November 2013. Currently, CET is still in the pilot phase and only targets CO_2_ emissions. The main transaction object is CO_2_ emissions quotas. The most important participants in the trading are the key emission enterprises for which direct and indirect CO_2_ emissions generated by fixed facilities together reached more than 10 thousand tons in the administrative area of Beijing. The other enterprises can also take part in the trading. The CET in Beijing includes five parts: CO_2_ emissions reporting by enterprises, CO_2_ emissions verification by third-party institutions, allocation and management of the CO_2_ emissions allowance by the government, CO_2_ emissions allowance trading by enterprises, and CO_2_ emissions allowance liquidation (compliance and offsets) by enterprises. Of the above five parts, the allocation of the CO_2_ emissions allowance is the most significant one related to the fairness and sustainability between enterprises. For existing facilities, the allowance for the next period of performance is given by the average of the CO_2_ emissions generated in the past 4 years by the enterprises and the emissions control coefficients made by the government for each industry (Table 12). For new facilities, the allowance for the next period of performance is given by the activity indicators of the new facilities such as the production or construction area and the advanced intensity of CO_2_ emissions in the industry to which the enterprises belong.

**Table 12:** Annual emissions control coefficients for different industries

	2013 (%)	2014 (%)	2015 (%)
Manufacturing and other industrial enterprises	98.0	96.0	94.0
Service enterprises	99.0	97.0	96.0
Gas facilities of thermal power plants	100.0	100.0	100.0
Coal-fired facilities of thermal power plants	99.9	99.7	99.5
Gas facilities of heating enterprises	100.0	100.0	100.0
Coal-fired facilities of heating enterprises	99.8	99.5	99.0

Table 12 indicates that the greatest pressure on CO_2_ emissions reduction is applied to the manufacturing enterprises, the second largest pressure is given to service enterprises, while the smallest pressure is given to the gas facilities of power plants and heating enterprises and the second smallest pressure is given to the coal-facilities of power plants and heating enterprises. There is a consensus between our study and that of the government that the electricity and heat supply sector plays a supportive role in the development of other sectors and should be focused on substituting clean energy for coal as the source of electricity and heat production. However, there may be a gap in the emissions control coefficients that would allow greater specification for the industrial sectors; this would enable the pressure for CO_2_ emissions reduction to be adjusted between the manufacturing sectors and the service sectors. This is based on the following two reasons: First, the emissions control coefficients are not based on the role played by each specific sector, which leads to unfair treatment of the internal sectors belonging to the manufacturing industry or the service industry; second, 54.17 % of the sectors belonging to the manufacturing industry (sector 2–22, 24–26) and 85.71 % of the sectors belonging to the service industry (sector 27–40) were takers in the CO_2_ emissions, which are the primary focus.

### Impacts of Chinese energy policies on GHG emissions

In order to achieve the Intended Nationally Determined Contribution (INDC) targets by 2020 and 2030, a series of energy policies have been put forward by the Chinese government on renewable energy, industrial structure and energy efficiency, shown as Table 13. Under these current policies, the Chinese energy-related CO_2_ emissions will be 9445 million tons by 2020 (IEA 2015), which are at the same level as the pledge the Chinese government committed to in 2009 (den Elzen et al. 2016). Many other studies also indicated that China would meet its 2020 carbon target commitment (Cansino et al. 2015; Jiang et al. 2013; Zhang et al. 2014a, b).

**Table 13:** Overview of the current policies in China. *Sources*: den Elzen et al. (2016), IEA (2015), Li and Yao (2009)

Sector	Policy/measure	Target
Energy supply	Medium and long term development plan for renewable energy	Increasing the share of gas in total primary energy supply to 10 % by 2020
		Limiting coal consumption to a maximum of 4.2 billion tonnes from 2020 onwards (coal cap)
		11.4 % share of non-fossil fuels (including nuclear) in primary energy consumption by 2015
	Updates for renewable energy capacity in 12th five-year plan	
	Renewable electricity	700 GW renewable electricity by 2020 (420 GW hydropower, 200 GW wind, 50 GW solar, 30 GW biomass, 0.1 GW tidal by 2020
	Solar hot water	800 million m^2^ collector area by 2020
	Biofuel	10 million tonnes ethanol, 2 million tonnes biodiesel by 2020
	Nuclear energy	58 GW nuclear energy by 2020 and 150 GW by 2030
Transport	Subsidies for hybrid and electric vehicles, biofuel target	Ethanol blending mandates 10 % in selected provinces
	Fuel efficiency standard	5 l/100 km for new cars (20 km/l) by 2020
Building	Promoting renewable energy utilization	15 % share of renewable energy in total energy consumption by 2020
Industry	Energy efficiency: Top 10,000 energy-consuming enterprises programme	Energy saving targets for energy-intensive industries, to be achieved by 2015. The target for steel production is 25 %, for the non-ferrous metal industry 18 %, and for cement production 3 %

Before climate conference in Paris in December 2015, China had formally submitted its climate pledge, including: (1) a peak in carbon emissions by 2030 or earlier; (2) a continued carbon intensity reduction target reaching 60–65 % below 2005 levels by 2030; (3) an increase in non-fossil energy sources to represent at least 20 % of total energy by 2030; and (4) a target to increase the forest stock volume by around 4.5 billion cubic meters on the 2005 level. However, if China does not propose new energy and climate policies, the climate pledge cannot be achieved. According to world energy outlook 2015 (IEA 2015), under the current polices scenario, the energy-related CO_2_ emissions will be 10,939 million tons by 2030 and 11,732 million tons by 2040, which indicate that a CO_2_ emissions peak cannot be reached by 2030. Moreover, the share of non-fossil energy in the total energy consumption will be 15.84 %—lower than the above climate pledge by 2030 (IEA 2015).

Furthermore, according to the Paris Agreement, governments should reduce greenhouse gas emissions to achieve a long-term goal of keeping the increase in the global average temperature to well below 2 °C above pre-industrial levels. This cannot be reached under the current policies. Following the existing policies for all the countries, the increase in the global average temperature will be 3.6 °C by 2100 above pre-industrial levels (IEA 2015). If the rise in the long-term average global temperature is limited to 2 °C, the energy-related CO_2_ emissions should be controlled to 6079 million tons by 2030 and 3318 million tons by 2040, only accounting for 55.57 and 28.28 % of the predicted emissions by 2030 and 2040, respectively, under the current policies scenario (IEA 2015). Therefore, new energy and climate policies need to be proposed and implemented (den Elzen et al. 2016; Liu 2012).

As the capital and one of the largest megacities in China, Beijing promised to reach the CO_2_ emission peak earlier than other regions in China, which makes a great contribution to the Chinese climate pledge achievement. Therefore, new energy policies should be considered and implemented. According to our analysis, new energy policies should be given to major sectors that emit a large amount of GHG in Beijing, including construction, transportation and electricity production sectors. Based on the past experiences on CO_2_ emission reduction of the Chinese and Beijing government, Beijing will become the first region to achieve the CO_2_ emission peak before 2030, which is attributed to renewable energy supply increasing, fuel efficiency in transport improving, building efficiency improving, and smaller emission cap in the CET. However, most current Chinese energy policies focus on CO_2_ emissions while ignore CH_4_ and N_2_O emissions. According to the Paris Agreement, we also should control CH_4_ and N_2_O emissions because they are more effective at trapping heat and have greater GWPs than CO_2_ (Zhang et al. 2015). Therefore, it can be extremely essential for the CH_4_ and N_2_O emissions in Beijing to reduce coal consumption, manage coal mining activities and waste generation.

### Impacts of Chinese energy policies on CET

**Fig. 6:**
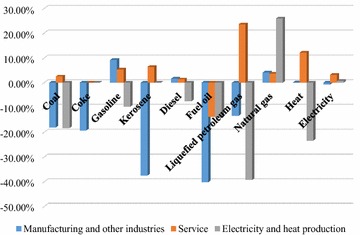
Energy consumption variations in different sectors between 2013 and 2014 in Beijing (%). *Data source*: BMBS (2015)

The impacts of energy policies on CET mainly reflect in the volume and price. Under the current energy policies proposed by the Chinese and Beijing government, energy consumptions in the industries existed in different variations between 2013 and 2014 in Beijing, shown as Fig. 6. In order to analyze the impacts of energy policies on CET, the means of classifying industries in Fig. 6 is the same as in Table 12.

Generally, a decrease occurred in most kinds of energy consumption in the electricity and heat production while an increase happened in all kinds of energy except fuel oil in the service. Though there had an increase in five types of energy consumption in the manufacturing and other industries, the rate of decrease was higher in the other five types of energy consumption. The above variations were consistent with the regulation degrees of energy policies. For example, the governments encourage natural gas as a substitute for coal in the power generation, as Tables 12 and 13 indicated. Therefore, the largest increase existed in the natural gas consumption in the electricity and heat production while a great decrease occurred in the coal consumption. As for the service, current energy policies attach more importance to transport than to other services, which led to an increase in most types of energy consumptions, and furthermore, an increase in CO_2_ emissions in the service.

The above variations in the energy consumptions affected the Beijing’s CET. First, the CET volume of the service was larger than that of other industries. According to Annual Report of Beijing Carbon Market 2014 (Zou et al. 2015), the CET volume of the service accounted for 40.92 %, which was larger than 22.09 % for the manufacturing and other industries and 13.65 % for the heat and electricity production. Second, the carbon price in 2015 was lower than that in 2014. The carbon price in 2015 was 46.69 CNY per ton, which was lower than 59.48 CNY per ton in 2014. This maybe because most kinds of fossil energy consumption decreased by more than 10 % while the CO_2_ emission control coefficient only decreased by 2 %. Therefore, the recent energy policies have promoted CO_2_ emissions reduction. After the pilot in 2014–2015, the Beijing’s CET should be more detailed for sectors rather than for the current three kinds of industries. In addition, flexibility for more auctions is needed, as well as rigorousness for the cap if new energy policies introduced to Beijing in order to comply with the Paris Agreement.

## Conclusions and policy implications

### Conclusions

In this paper, an input–output model has been developed to demonstrate the roles of sectors in the GHG emissions of the production system in Beijing. The input–output model first identifies four components including the own, feed-back, spillover and intra-sector spillover components by capturing the economic and environmental relationships that exist among the production activities. Then, we developed an emissions matrix with the above four components to analyze the pull and push effects of sectors and to determine the sectoral roles in the CO_2_, N_2_O and CH_4_ emissions, for both the giver and taker. Furthermore, after identifying the roles played by the sectors, we more importantly address the implications for policies on energy use and the current CET system.

With the aid of the input–output model, we found that direct emissions are concentrated in a few sectors. The manufacturing sectors were the major emitters in CO_2_ and N_2_O pollutions, while the service sectors were the most important participators in CH_4_ emissions. However, the total emissions generated by the final demand of sectors exhibited very different patterns. Services played a leading role in these three GHG emissions. This is because a large amount of direct emissions generated by the manufacturing sectors were caused by delivering intermediate goods to meet the final demand of services. These results suggest that sectoral emissions reduction policies should take into account the trade in intermediates in the production system.

In addition, the pull and push effects of the sectors were investigated, and the roles played by the sectors in GHG emissions were identified. Strikingly similar results are found for these three GHG emissions in that the largest takers were the sectors Construction, Scientific studies and technical services and Finance. Not surprisingly, as the basis and support of the whole economy, the sectors Production and distribution of electricity, gas and water and Transportation, storage, posts and telecommunications were the largest givers for these three GHG emissions. Therefore, services may need more regulations on emissions reduction than the manufacturing sectors. In addition, electricity production and transportation should not have restraints on emissions based on output but should focus on adopting clean energy sources (e.g., hydropower, wind power and biomass power) and carbon capture and storage technology to reduce the intensity of GHG emissions.

### Policy implications for energy utilization

Above and beyond our understanding of the sectoral roles in GHG emissions, certain implications can be extracted from the above results which are interesting in policy terms on energy utilization:

First, with respect to the takers, the government should give some financial support and guidelines for technical innovation in reducing consumption of fossil fuels and electricity. Under the current CET in Beijing, though improving the production technology to reduce energy use is the driving force to control production cost, the enterprises belonging to the sectors defined as takers (e.g. most service sectors) would consider the balance between the R&D (Research & Development) investments on energy utilization technologies in the short term and the economic and environmental gains in the long term. Some small enterprises may not be able to afford a lot of R&D investment in the short run to improve the energy utilization technology. Therefore, policy makers should consider and implement energy taxes combined with policies concerning technology and relative prices to increase production while reducing energy consumption. Learning from the practices of EU members (e.g. Finland, Sweden and Norway), an appropriate policy that can be taken into account is reducing taxes on employment by revenue recycling while levying energy taxes in order to have environmental gain without adverse effects on production (Ge and Lei 2014).

Second, with respect to the givers, the government should provide financial support and policies regarding clean energy (e.g. ethanol as fuel for vehicles) large-scale production and use. For Production and distribution of electricity, gas and water, the following policy recommendations should be considered in Beijing: boosting the construction of large-scale as well as small hydropower plants; encouraging the construction of large capacity solar power generation systems; and promoting the development of power generation from forestry residuals, livestock excrement and municipal refuse (Yuan et al. 2014). For Transportation, storage, posts and telecommunications, policies on promoting hybrid cars and electric vehicles and fuel ethanol should be taken into account in energy conservation and reducing GHG emissions.

### Policy implications for CET

Some important policy implications for CET in Beijing may arise from our findings.

First, an absolute cap should be substituted for an intensity-based cap on CO_2_ emissions because of the uncertainty of the sectoral output due to the rapid economic development and accelerated structural adjustment in Beijing. Let us take services as an example to discuss this implication. The government has been promoting services development in Beijing because of the need for economic structural adjustment and also because the direct CO_2_ emissions of services are not high. However, the indirect CO_2_ emissions, i.e., the pull effect on the direct CO_2_ emissions of other sectors, especially sector 23 and sector 27, are very high, which is consistently neglected by us. To reduce CO_2_ emissions, the services industry should decrease the dependency on electricity production and transportation in the short term. This is very difficult for an industry that depends on growth. In addition, certain factors affect the market demand for goods and services, which leads to the uncertainty of sectoral output. For example, the market demand for the services from Hotel and restaurants (sector 30) is influenced by the ‘Eight-point Rules’ issued by the Political Bureau of the CPC (Communist Party of China) Central Committee. This would cause allowance waste. Therefore, an intensity-based cap may be more suitable than the current absolute cap in Beijing.

Second, under the intensity-based cap, the emissions control coefficients should be set for the sectors according to their roles in CO_2_ emissions. Currently, the emissions control coefficients are set according to the industrial classifications, including the agricultural industry, the manufacturing industry and the service industry. Under the current level of technology, there is relatively more pressure on the manufacturing sectors to reduce CO_2_ emissions, which is unfair because some of them such as the mining sectors support the development of other sectors. Moreover, though the pressure on services to reduce CO_2_ emissions is second to the manufacturing industry, the role of the service sectors is different. For example, because most of the manufacturing sectors and the service sectors are dependent on transportation, it is difficult for Transportation, storage, posts and telecommunications (sector 27) to reduce the direct CO_2_ emissions without updating the transportation equipment. In other words, the givers cannot reduce their output to meet the CO_2_ emissions control targets because they are the basis for the development of the majority of the sectors. Givers can meet their targets by improving technology and production equipment, which requires a great deal of capital investment. Therefore, the emissions control coefficients for the givers should be set for certain technical aspects or the equipment for the production process, taking the emissions control coefficients setting for thermal power plants and heating plants as references. The emissions control coefficients for the takers can be set according to their direct and indirect CO_2_ emissions. Without a doubt, another important factor that is residential demand for sectoral production and services should be taken into account to set emissions control coefficients. In addition to inter-industry demand, both for the givers or takers, the final demand of resident also affects their production and CO_2_ emissions. For instance, residents’ ownership of electrical appliance grows fast because rapid urbanization brought about lifestyle changes and economic development gave people higher incomes, which increases electricity consumption and furthermore pulls up electricity production. For the same reason, lifestyle change and higher income also promote residents’ more demand for services. Therefore, the emissions control coefficients should also consider the residential demand change for sectoral production and services.

Third, auctions should be introduced into the allowance allocation for takers. In China, Guangdong is the first and currently the only pilot province to introduce auctions into the allowance allocation. After the first pilot year (July 2013–July 2014), the compliance rate of enterprises and the allowance on CO_2_ emissions reduction achieved 98.90 and 99.97 %, respectively. In the Guangdong industrial sectors, the enterprises must acquire a 3 % allowance through auction, and they can then activate a 97 % allowance for free; for large public buildings, 100 % are allocated for free by benchmarking before the compliance period. At present, the government-allocated allowance for the enterprises is free in Beijing. However, the CET will eventually be completely adjusted by the market, as is the case for CET in the EU (European Union). Specifically, a certain level of auction, such as 3 %, can be introduced to the allowance for the takers; and a lesser amount for auction, which should be smaller than the proportion of auction in the allowance for the takers, can be introduced to the allowance for the givers; alternatively, 100 % can be free for the allowance of the givers.

Fourth, N_2_O and CH_4_ emissions should be included in the CET of Beijing as most sectors played the same role in CO_2_, N_2_O and CH_4_ emissions. There are forty sectors included in this study, of which eight sectors played a different role in CO_2_, N_2_O and CH_4_ emissions (sectors 1, 3, 7, 11, 12, 15, 29 and 30), nine sectors played the same role, namely, that of giver, in these three types of GHG emissions (sectors 4, 5, 10, 13, 22, 23, 24, 27, and 36), and the remaining twenty-three sectors played the same role, namely, that of taker, in the above three types of GHG emissions. For the sectors that played different roles in CO_2_, N_2_O and CH_4_ emissions, policy makers have two approaches to allocate allowance: one way is to set different emissions control coefficients for the enterprises according to the sectoral roles played in CO_2_, N_2_O and CH_4_ emissions; the other way is to set one emissions control coefficient for the enterprises according to the sectoral role played in the GWPs. For the sectors that exhibited the same role in CO_2_, N_2_O and CH_4_ emissions, policy makers may consider extending the above policy implications 1–3 for CO_2_ CET to N_2_O and CH_4_ emissions.

## Appendix

See Tables 14 and 15.

**Table 14:** Sectoral information for input–output extension table of Beijing for 2010

Sector code	Sector category
1	Farming, forestry, animal husbandry and fishery
2	Mining and washing of coal
3	Extraction of petroleum and natural gas
4	Mining and processing of metal ores
5	Mining and processing of nonmetal ores and other ores
6	Manufacture of foods and tobacco
7	Manufacture of textile
8	Manufacture of textile wearing apparel, footware, caps, leather, furs
	and related products
9	Manufacture of wood and furniture
10	Manufacture of paper, printing and manufacture of aticles for culture, education and sports activity
11	Processing of petroleum, cokiing, processing of nuclear fuel
12	Chemical industry
13	Manufacture of non-metallic mineral products
14	Smelting and pressing of metals
15	Manufacture of metal products
16	Manufacture of general and special purpose machinery
17	Manufacture of transport equipment
18	Manufacture of electrical machinery and equipment
19	Manufacture of communication equipment, computers and other electronic equipment
20	Manufacture of measuring instruments and machinery for culture activity and office work
21	Manufacture of artwork and other manufacturing
22	Recycling and disposal of waster
23	Production and distribution of electricity and heat
24	Production and distribution of gas
25	Production and distribution of water
26	Construction
27	Transportation, storage, posts and telecommunications
28	Information transmission, computer services and software
29	Wholesale trade and retail trade
30	Hotel and restaurants
31	Finance
32	Real estate trade
33	Tenancy and commercial services
34	Scientific studies and technical services
35	Water, environment and municipal engineering conservancy
36	Resident services and other services
37	Education
38	Health care, social security and social welfare
39	Culture, art, sports and recreation
40	Public manage and social organization

**Table 15:** Global warming potentials for CO_2_, N_2_O and CH_4_ emissions over the horizon of 100 years

GHGs	GWP_100_ in AR5
CO_2_	1
N_2_O	265
CH_4_	28
